# The Risk of Type 2 Diabetes in Men Is Synergistically Affected by Parental History of Diabetes and Overweight

**DOI:** 10.1371/journal.pone.0061763

**Published:** 2013-04-22

**Authors:** Cecilia Wikner, Bruna Gigante, Mai-Lis Hellénius, Ulf de Faire, Karin Leander

**Affiliations:** 1 Division of Cardiovascular Epidemiology, Institute of Environmental Medicine, Karolinska Institutet, Stockholm, Sweden; 2 Department of Cardiology, Karolinska University Hospital, Stockholm, Sweden; 3 Institute of Medicine, Karolinska Institutet, Stockholm, Sweden; German Diabetes Center, Leibniz Center for Diabetes Research at Heinrich Heine University Duesseldorf, Germany

## Abstract

Interactions between genetic- and lifestyle factors may be of specific importance for the development of type 2 diabetes. Only a few earlier studies have evaluated interaction effects for the combination of family history of diabetes and presence of risk factors related to lifestyle. We explored whether 60-year-old men and women from Stockholm with a parental history of diabetes are more susceptible than their counterparts without a parental history of diabetes to the negative influence from physical inactivity, overweight or smoking regarding risk of developing type 2 diabetes. The study comprised 4232 participants of which 205 men and 113 women had diabetes (the vast majority type 2 diabetes considering the age of study participants) and 224 men and 115 women had prediabetes (fasting glucose 6.1–6.9 mmol/l). Prevalence odds ratios (OR) with 95% confidence intervals (95% CI) were calculated using logistic regression. Biologic interaction was analyzed using a Synergy index (S) score. The crude OR for type 2 diabetes associated with a parental history of diabetes was 2.4 (95% CI 1.7–3.5) in men and 1.4 (95% CI 0.9–2.3) in women. Adjustments for overweight, physical inactivity and current smoking had minimal effects on the association observed in men whereas in women it attenuated results. In men, but not in women, a significant interaction effect that synergistically increases the risk of developing type 2 diabetes was observed for the combination of BMI>30 and a parental history of diabetes, S 2.4 (95% CI 1.1–5.1). No signs of interactions were noted for a parental history of diabetes combined with physical inactivity and smoking, respectively. In conclusion, obesity in combination with presence of a parental history of diabetes may be particularly hazardous in men as these two factors were observed to synergistically increase the risk of developing type 2 diabetes in men.

## Introduction

Type 2 diabetes is a major public health problem in both developing- and industrialized countries [1]. The disease is chronic and may cause suffering due to severe complications related to micro- and macrovascular pathology affecting several organs. Furthermore, its status as a strong risk factor for cardiovascular disease is well known [2]. Prediabetes is an intermediate stage in between normal glucose regulation and diabetes and can be characterized by either impaired fasting glucose (IFG) or impaired glucose tolerance (IGT) [1]. The current knowledge about the etiology of diabetes and prediabetes is insufficient. Previous studies suggest that genetic factors as well as an array of different lifestyle factors are associated with the onset of type 2 diabetes [3], [4], [5]. The exact significance of individual genes for disease onset is still only partly resolved although previous twin studies applying quantitative genetic models suggested a substantial genetic component behind this disease [6]. Recent genome-wide association studies have identified 12 new independent loci associated with type 2 diabetes [7] and at present a total of about 50 loci associated with type 2 diabetes have been identified [8], [9]. Combined, these loci however only account for about 10% of the observed familial clustering in Europeans [8]. A hereditary component is also suggested from a number of studies that assessed the influence of a family history of diabetes on risk of type 2 diabetes; most studies report a two- to six fold increased relative risk of type 2 diabetes and the associations appear to be independent of lifestyle factors [10], [11]. Results from earlier studies are however not consistent regarding the magnitude of sex specific associations [12], [13], [14].

Overweight and lifestyle related risk factors such as physical inactivity, active smoking and dietary habits have established effects on type 2 diabetes [3], [4], [5]. Further, metabolic factors such as lipid levels or hypertension are often considered in the risk assessment [15]. Reports about gender differences regarding influence from lifestyle factors are scarce. It has been hypothesized that interactions between genetic- and environmental factors are of specific importance for the development of type 2 diabetes. A number of studies have aimed to unravel such interactions [16], however with limited success. Although a family history of diabetes may reflect both genetic susceptibility and exposures to environmental factors that are shared within the family, an observed association between family history and risk of type 2 diabetes, that remains after adjustments for established lifestyle-related factors may be interpreted as an indicator of genetic susceptibility. Analyses of interactions between family history and different environmental factors may thus identify environmental factors that if combined with a genetic susceptibility are particularly hazardous with regard to diabetes. Only a few earlier studies have evaluated interaction effects for the combination of family history of diabetes and presence of risk factors related to lifestyle and results are divergent [12], [17].

We assessed whether parental history of diabetes is associated with risk of type 2 diabetes or prediabetes using baseline data from a cohort of 60-year-old men and women from Stockholm. We also explored if those with a parental history of diabetes are more susceptible (as compared to those without a parental history) to the negative influence of physical inactivity, overweight or smoking on the risk to develop diabetes.

## Materials and Methods

The Ethical Committee at Karolinska Institutet, here referred to as the Institutional review board (IRB), approved the design of the present cohort study in 1996 (registration number: 96–398). All study participants gave their informed oral consent to be enrolled in the study. Written consent was not collected because at the time the study was initiated forms for written consent were not in current use. The IRB has in several more recent matters approved continued research on the current material (e.g. matters with registration numbers 99–306, 03–100 and 03–115), with reference to the fact that eligible men and women were already informed (in written form) about the study and that participation was voluntary. Those who decided to participate, after having received information about the study, were asked to contact a booking central by telephone for making an appointment to attend a physical examination. All clinical investigations were conducted according to the principles expressed in the Declaration of Helsinki. The IRB approved this consent procedure (96–398).

An invitation letter was sent by mail to eligible subjects informing about the study. The information included description of rationale for the study, study aims, study design, and that participation involved attending a health investigation plus filling out a questionnaire. Details about measurements to be performed at the health investigation were given as well. Results were to be interpreted by a physician and delivered to participants, with medical advice if needed. The letter also stated that participation was voluntary. The receiver of the invitation was asked kindly to contact a booking central to inform whether he or she would like to participate or not. The nurses working at the booking central documented each reply, so that those who agreed to participate was appointed a time for investigation, and those that were not willing to participate were withdrawn from the list of eligible participants. A second letter of invitation, a reminder, was sent to those who did not reply to the first letter, and the same procedure for documentation of oral consent was used. Instead of calling, some of the individuals invited replied by sending a letter to the booking central declining participation. Some never responded.

The recruitment of study participants started July 1^st^ 1997 and ended June 30^th^ 1998. Every third man and woman who turned 60 during the time period of recruitment and who at that time lived in Stockholm County was randomly selected from the Swedish population register and invited to participate in the study. In total 5460 subjects, 2779 men and 2681 women, were invited, out of which 4232 individuals participated; 2039 men (73%) and 2193 women (82%).

The study participants underwent a health examination and filled out a questionnaire about, among other things, earlier diseases, diseases in relatives, lifestyle and the use of medications. During the health examination the participantś weight and height in standing position without shoes were measured. As an indicator of abdominal adiposity, the sagittal abdominal diameter (SAD) was measured as previously described [18]. The waist- and hip circumferences were also measured.

Venous blood samples from an antecubital vein were drawn after overnight fasting since 24∶00 the night before. The samples were incubated at −70° Celsius. Some of the parameters measured in serum were glucose, insulin, triglycerides, High Density Lipoprotein (HDL) cholesterol and Low Density Lipoprotein (LDL) cholesterol. LDL cholesterol was calculated using Friedewalds formula, serum glucose was measured with an enzymatic colorimetric test (Bayer Diagnostics, Tarrytown, NY, USA) and serum insulin levels were determined with the ELISA technique (Boehringer Mannheim GmbH, Diagnostica, Germany).

### Definition of Study Outcome

Study participants were classified as suffering from diabetes if 1) they in the questionnaire indicated presence of diabetes, or 2) fasting serum glucose level (data from one measurement) was ≥7.0 mmol/l (cut-off value stipulated by the World Health Organization [1], or 3) they in the questionnaire reported use of any medication classified as antidiabetics according to the Pharmaceutical Specialities in Sweden (FASS) 1998, codes under A10.

Prediabetes was identified according to prevailing IFG diagnostic criteria, i.e. fasting glucose levels within the range 6.1–6.9 mmol/l [1]. Only individuals not reporting presence of diabetes or intake of medication for diabetes were considered.

### Classification of Exposures

Parental history of diabetes was defined as presence of diabetes in either the mother or the father (or in both) according to questionnaire data filled in by the participants. There were no questions about type of diabetes in the parent. Individuals not indicating presence of a parental history of diabetes were included in the reference category.

Participants who in the questionnaire indicated that they smoked regularly were classified as current smokers and those who stated that they previously had been smoking regularly were classified as ex-smokers. For the interaction analyses current smokers were classified as exposed whereas never-smokers and ex-smokers formed the reference category.

Level of leisure time physical activity during the past year was measured using a question that was later on successfully validated by the Swedish National Food Administration, report 21, 2004, using as gold standard an accelerometer measuring the level of physical activity. Four different answer alternatives were predefined: 1) Sedentary; exercise less than 2 hours a week; 2) Light exercise; exercise at least 2 hours a week without sweating; 3) Moderate regular exercise; at least 30 minutes of exercise 1–2 times a week that makes you sweat; and 4) Regular exercise and training; at least 30 minutes of exercise that makes you sweat, 3 times a week or more. For use in the interaction analyses a dichotomized variable was created with those who had answered with alternative 1 classified as exposed and those who had answered with alternative 2, 3 or 4 classified as non-exposed.

The participants were also asked about work related physical activity. To the question “How big part of your working day do you perform sedentary work?” there were four answer alternatives: 1) Sedentary almost all day; 2) Sedentary about half of the day; 3) Sedentary less than half of the day; and 4) Not sedentary at all. In the interaction analyses those who had answered with alternative 1 were classified as exposed and those who had answered with alternative 2, 3 or 4 were classified as non-exposed.

From data on height (cm) and weight (kg) collected at the health examination the body mass index (BMI; kg/m^2^) was calculated. For the interaction analyses the BMI variable was dichotomized. Two different cut off values were used: BMI>25 (overweight) and BMI>30 (obesity). From data on waist- and hip circumference (cm), waist/hip ratios (WHR) were calculated. From the distribution of SAD values (cm), WHR values and waist circumference values, cut-off limits were chosen that corresponded to the 90^th^ percentile values in men and women respectively: SAD 25.0 cm and 23.5 cm; WHR 1.03 and 0.92; waist circumference 111 cm and 102 cm.

### Statistical Analysis

P-values for differences in the distribution of different variables when comparing individuals without diabetes with those with diabetes and prediabetes, respectively, were calculated using Chi-square analysis, the Student´s t-test and the Kruskal-Wallis test as appropriate. All p-values were two-sided and level of significance was set to 0.05.

Prevalence Odds ratios (OR), with 95% confidence intervals (CI), of diabetes were calculated using logistic regression. The OR should approximate incidence rate ratios in the population under study considering that the required assumptions for such approximation [19] are reasonably met; there was only a small increase in the prevalence of type 2 diabetes among the Swedish population during the period 1997–2003, from 2.2% to 3.5% [20], and the duration of the disease is most likely the same in individuals with and without a parental history. Crude analyses as well as analyses with single and multiple adjustments were performed. Smoking, overweight and physical inactivity were adjusted for by means of dummy variables.

We investigated ‘biological interactions’ as defined by Rothman [21]. Interactions between a parental history of diabetes and each of the three selected risk factors smoking, overweight and physical inactivity were analysed using Synergy index (S) scores [22], [23]. The S score is defined as equal to [OR_11_−1]/[(OR_01_−1)+(OR_10_−1)] where OR_11_ is the OR for diabetes associated with the exposures combined whereas OR_10_ and OR_01_ are ORs of diabetes associated with the single exposures (in absence of the other single exposure). All ORs are calculated using as reference category those non-exposed to each of the single exposures. Thus, the S score indicates if the risk in double exposed is higher than expected based on the assumption of an additive effect exerted by the single exposures. An S score exceeding 1.0 indicates interaction and an S score below 1.0 indicates an antagonistic effect.

The software SAS 9.2 (SAS Institute, Cary, NC, USA) was used for all statistical analyses.

## Results

Out of the 4232 cohort participants, one man and two women were excluded from the analyses because they had no glucose value registered and they had left the question about presence of diabetes blank. In total, 205 men (10.1%) and 113 women (5.2%) were classified as having diabetes. Out of these, 93 men and 41 women had not self-reported diabetes but were identified due to elevated glucose value. Out of the male participants with self-reported diabetes, 78% reported intake of medication classified as antidiabetics. The corresponding proportion in women was 71%. The most commonly reported medications were those containing glibenklamid (34%), insulin (31%) and metmorfin (15%). In 3% of men reporting diabetes, and in 13% of women, the reported diabetes could not be validated by either glucose value or reported intake of diabetes medication. The number of men and women identified with prediabetes was 224 and 115, respectively.

In men, but not in women, a parental history of diabetes was significantly more frequent among individuals with diabetes or prediabetes than among individuals in the comparison group (table 1). The difference in men was mainly driven by a different proportion of a maternal history of diabetes (table 1). Excluded from the analyses of a maternal history of diabetes are individuals who reported presence of diabetes in the father, and vice versa regarding the analyses of a paternal history of diabetes. A biparental history of diabetes was reported by 25 women and 14 men; among these, one woman and seven men had diabetes.

**Table 1:** Characteristics of study participants by categories of diabetes, prediabetes and reference category (neither diabetes nor prediabetes).

	Men					Women				
	Diabetes (n = 205)	*P*	Prediabetes (n = 224)	*p*	Reference category (n = 1609)	Diabetes (n = 113)	*p*	Prediabetes (n = 115)	*p*	Reference category (n = 1963)
Parental history of diabetes %	22.4	<0.0001	16.5	0.02	11.2	20.4	0.19	16.5	0.81	15.7
Maternal history of diabetes	14.2	<0.0001	9.4	0.07	6.2	11.5	0.51	11.3	0.56	9.6
Paternal history of diabetes	4.9	0.83	6.7	0.16	4.5	8.0	0.14	4.4	0.81	4.8
Smokers %	17.0 (^m^17)	0.82	23.0 (^m^15)	0.40	20.8 (^m^59)	27.6 (^m^8)	0.24	32.1 (^m^3)	0.034	21.4 (^m^79)
Ex-smokers %	53.7 (^m^17)	0.07	45.5 (^m^15)	0.67	45.4 (^m^59)	27.6 (^m^8)	0.61	25.0 (^m^3)	0.46	32.3 (^m^79)
Physical inactivity leisure time %	12.5 (^m^21)	0.32	13.6 (^m^11)	0.12	10.1 (^m^60)	19.4 (^m^10)	0.01	21.6 (^m^4)	0.001	11.2 (^m^75)
Physical inactivity at work %	28.7 (^m^20)	0.30	30.6 (^m^15)	0.61	32.4 (^m^62)	32.4 (^m^11)	0.85	37.6 (^m^6)	0.35	33.3 (^m^94)
BMI>25%	83.9	<0.0001	79.0	<0.0001	65.8	84.1	<0.0001	73.0	0.001	57.2
BMI>30%	38.5	<0.0001	31.3	<0.0001	14.6	46.0	<0.0001	36.5	<0.0001	17.7
Born in Sweden %	73.7 (^m^15)	0.37	79.0 (^m^10)	0.83	80.2 (^m^38)	70.8 (^m^5)	0.06	75.7 (^m^3)	0.34	79.1 (^m^53)
Level of education:										
University %	22.2 (^m^16)	0.03	24.2 (^m^13)	0.09	29.8 (^m^42)	20.6 (^m^6)	0.09	24.1 (^m^3)	0.36	28.1 (^m^58)
Compulsory/Secondary school %	77.8		75.8		70.2	79.4		75.9		71.9
BMI (kg/m^2^)	29.2±4.3	<0.0001	28.4±4.03	<0.0001	26.5±3.6	30.3±5.7	<0.0001	28.9±6.0	<0.0001	26.3±4.3
Waist-to-hip ratio	0.99±0.06	<0.0001	0.97±0.06	<0.0001	0.94±0.06 (^m^3)	0.88±0.07	<0.0001	0.86±0.07	<0.0001	0.82±0.06 (^m^1)
Waist circumference	104.2±11.8	<0.0001	101.5±11.0	<0.0001	96.4±9.8 (^m^2)	96.7±13.1	<0.0001	93.3±14.9	<0.0001	85.6±11.3 (^m^1)
Sagittal abdominal diameter (cm)	23.0 (21.0–25.0)	<0.0001	22.5 (20.5–24.5) (^m^1)	<0.0001	21.0(19.3–22.6) (^m^3)	21.9 (19.8–24.5) (^m^1)	<0.0001	21.0 (19.5–23.5)	<0.0001	19.4 (17.7–21.0) (^m^2)
Glucose (mmol/l)	8.7 (7.4–11.1)	<0.0001	6.3 (6.2–6.6)	<0.0001	5.2(4.9–5.5)	8.3 (7.1–12.3)	<0.0001	6.3 (6.1–6.5)	<0.0001	5.0 (4.7–5.3)
Insulin (µU/ml)	15.3 (10.3–23.9)	<0.0001	11.6 (8.6–17.6)	<0.0001	8.6(6.4–11.8)	14.6 (10.9–20.9) (^m^1)	<0.0001	10.6 (8.5–14.9)	<0.0001	8.3 (6.2–11.0)
HDL (mmol/l)	1.1 (0.9–1.4)	<0.0001	1.2 (1.0–1.5)	0.01	1.3(1.1–1.5)	1.4 (1.2–1.7)	<0.0001	1.5 (1.3–1.7)	<0.0001	1.6 (1.4–1.9)
LDL (mmol/l)	3.6±0.9 (^m^16)	0.002	3.8±1.0 (^m^13)	0.41	3.9±0.9 (^m^19)	3.9±1.0 (^m^3)	0.74	4.2±1.03 (^m^2)	0.001	3.9±0.9 (^m^8)
Triglycerides (mmol/l)	1.7 (1.2–2.4)	<0.0001	1.4 (1.0–2.1)	<0.0001	1.1(0.8–1.6)	1.5 (1.0–2.3)	<0.0001	1.3 (1.0–1.9)	<0.0001	1.1 (0.8–1.4)
Diastolic blood pressure (mmHg)	90.2±10.7 (^m^1)	<0.0001	90.6±10.8 (^m^1)	<0.0001	86.8±10.3 (^m^2)	83.0±9.8	0.05	86.0±11.03	<0.0001	81.2±9.7 (^m^1)
Systolic blood pressure (mmHg)	151.5±20.3 (^m^1)	<0.0001	149.0±21.5 (^m^1)	<0.0001	140.9±20.1 (^m^2)	143.0±23.5	<0.0001	144.3±23.0	<0.0001	133.1±21.6 (^m^1)

Continuous data are expressed as mean ± standard deviation or median with the interquartile range within parenthesis.

(^m^) indicates number of individuals with missing value.

*p*-value for current smokers and ex-smokers, respectively, calculated using never smokers as reference category.

As compared to the reference group, men and women with diabetes and prediabetes, respectively, presented significantly higher levels of glucose and insulin, and also higher values of BMI, SAD, blood pressure, LDL cholesterol and triglycerides; HDL values were significantly lower (table 1). Women with diabetes or prediabetes more frequently than the reference group reported a physically inactive leisure time. They also more frequently reported current smoking (table 1).

Among men reporting a parental history of diabetes (exposed), the proportion identified with diabetes and prediabetes was 20.4% and 17.1%, respectively. In men with no parental history of diabetes (unexposed) the corresponding proportions were 10.0% and 11.6%. Current smoking was less common in exposed men than in unexposed: 21.4% as compared to 15.7%. The mean BMI in exposed men was 27.4±3.9 and in unexposed it was 26.9±3.8 (p<0.0001). Among the exposed, 17.4% reported presence of diabetes in a sibling as compared to 6.2% among non-exposed. Among exposed women, the proportion identified with diabetes and prediabetes was 7.0% and 5.8%, respectively. The corresponding proportions in unexposed were 5.2% and 5.5%. Current smoking was equally frequent (about 22%) comparing the two exposure groups, whereas BMI was higher in the exposed group: 27.7±4.7 vs. 26.5±4.6. Among the exposed women, 13.7% reported presence of diabetes in a sibling as compared to 3.6% among non-exposed.

In men, the crude OR of diabetes associated with a parental history of diabetes was 2.4 (95% CI 1.7–3.5) (table 2); for prediabetes it was 1.6 (95% CI 1.1–2.3). In women, the corresponding ORs were 1.4 (95% CI 0.9–2.3) and 1.1 (95% CI 0.6–1.8), respectively. The OR of diabetes after adjustments for smoking, leisure time physical activity, physical activity at work and BMI was very similar to the crude value. In women, however, the adjusted OR was somewhat lower as compared to the crude value. A maternal history of diabetes in men was more strongly associated with diabetes risk than a paternal history of diabetes. A tendency of the opposite was noted in women. A family history, defined as presence of diabetes in a parent or in a sibling, was associated with presence of diabetes both in men and women.

**Table 2:** Prevalence odds ratios of diabetes and prediabetes associated with a parental history of diabetes.

	Men		Women		Both sexes	
	OR crude	OR adjusted^a^	OR crude	OR adjusted^a^	OR crude	OR adjusted^a^
Outcome: Diabetes						
Parental history of diabetes	2.4 (1.7–3.5)	2.4 (1.6–3.5)	1.4 (0.9–2.3)	1.1 (0.7–1.9)	1.8 (1.3–2.3)	1.8 (1.3–2.4)
Paternal history of diabetes	1.2 (0.6–2.4)	1.4 (0.7–2.9)	1.7 (0.9–3.6)	1.4 (0.7–3.1)	1.4 (0.9–2.3)	1.5 (0.9–2.4)
Maternal history of diabetes	2.6 (1.7–4.1)	2.6 (1.6–4.1)	1.3 (0.7–2.3)	1.1 (0.5–2.1)	1.8 (1.3–2.6)	1.8 (1.2–2.6)
Biparental history of diabetes	8.1 (2.8–23.3)	9.3 (3.0–29.2)	0.7 (0.1–5.4)	0.6 (0.1–4.2)	3.0 (1.3–6.5)	2.9 (1.2–7.0)
Family history of diabetes^c^	2.4 (1.7–3.4)	2.7 (1.8–3.8)	2.1 (1.4–3.2)	1.9 (1.2–2.9)	2.3 (1.8–3.0)	2.3 (1.8–3.1)
Outcome: Prediabetes						
Parental history of diabetes	1.6 (1.1–2.3)	1.6 (1.0–2.4)	1.1 (0.6–1.8)	1.0 (0.6–1.7)	1.3 (1.0–1.8)	1.3 (0.9–1.8)
Paternal history of diabetes	1.6 (0.9–2.8)	1.7 (0.9–3.0)	0.9 (0.4–2.3)	0.8 (0.3–2.1)	1.4 (0.9–2.4)	1.3 (0.8–2.1)
Maternal history of diabetes	1.6 (1.0–2.6)	1.5 (0.9–2.6)	1.2 (0.7–2.2)	1.1 (0.6–2.1)	1.4 (1.0–2.1)	1.3 (0.9–2.0)
Biparental history of diabetes	1.0 (0.1–8.4)	1.0 (0.1–8.6)	0.7 (0.1–5.3)	0.7 (0.1–5.0)	0.8 (0.2–3.5)	0.8 (0.2–3.3)
Family history ofdiabetes^c^	1.5 (1.0–2.1)	1.5 (1.0–2.2)	1.0 (0.6–1.6)	0.8 (0.5–1.4)	1.2 (0.9–1.7)	1.2 (0.9–1.6)

OR, prevalence odds ratio.

a Adjusted for body mass index, leisure time physical activity, physical activity at work and smoking.

b Adjusted for factors under a, with additional adjustment for sex.

c Family history of diabetes defined as presence of diabetes in either a parent or a sibling.

As shown in table 3, in men a BMI>30 combined with a parental history of diabetes produced an S score of 2.4 (95% CI 1.1–5.1). Corresponding analyses (in men) using as cut-off limits the sex specific 90th percentile values of SAD (25.0 cm), waist circumference value (111 cm), WHR (1.03) and BMI (31.7 kg/m^2^) produced S scores of 4.1 (95% CI 1.4–12.0), 2.8 (95% CI 1.1–7.1), 1.8 (95% CI 0.6–5.2) and 3.0 (95% CI 1.2–7.4), respectively. In women no corresponding indications of interactions were observed. Adjustments for physical activity and smoking had no substantial influence on the results (data not shown). The use of the “family history of diabetes”-definition in the analyses of interaction produced results in men that were less pronounced: The corresponding S scores to those listed above based on the 90^th^ percentile cut-off values were 2.6 (95% CI 1.0–6.7), 2.2 (95% CI 0.9–5.3), 1.7 (95% CI 0.6–4.5) and 2.2 (95% CI 1.1–4.4). In women, results were in essence the same using the alternative definition of a family history of diabetes.

**Table 3:** Interaction effects for parental history of diabetes in combination with high BMI, physical inactivity and smoking, respectively.

	Men				Women			
Exposure	Number of exposed cases	OR ofdiabetes	95% CI	S (95% CI)	Number of exposed cases	OR ofdiabetes	95% CI	S (95% CI)
BMI≤25 without PHD (reference)	26	1.0			14	1.0		
BMI>25 without PHD	133	2.7	1.8–4.2		76	4.4	2.5–7.9	
BMI≤25 with PHD	7	2.5	1.0–6.0		4	2.2	0.7–6.8	
BMI>25 with PHD	39	5.9	3.5–10.1	1.5 (0.7–3.3)	19	4.8	2.4–9.7	0.8 (0.4–1.8)
BMI≤30 without PHD (reference)	101	1.0			48	1.0		
BMI>30 without PHD	58	3.4	2.4–4.8		42	4.4	2.8–6.7	
BMI≤30 with PHD	25	2.0	1.2–3.2		13	1.6	0.8–3.0	
BMI>30 with PHD	21	9.1	5.0–16.6	2.4 (1.1–5.1)	10	4.0	2.0–8.3	0.8 (0.3–2.1)
Physical activity leisure time without PHD (reference)	120	1.0			62	1.0		
Physical inactivity leisure time without PHD	20	1.4	0.9–2.3		19	2.3	1.4–4.0	
Physical activity leisure time with PHD	41	2.5	1.7–3.7		21	1.7	1.0–2.8	
Physical inactivity leisure time with PHD	3	2.4	0.7–8.4	0.7 (0.1–6.6)	1	0.8	0.1–6.2	No result
Physical activity at work without PHD (reference)	102	1.0			54	1.0		
Physical inactivity at work without PHD	39	0.8	0.6–12		27	1.0	0.6–1.6	
Physical activity at work with PHD	30	2.5	1.6–4.0		15	1.4	0.8–2.5	
Physical inactivity at work with PHD	14	1.9	1.0–3.4	0.6 (0.1–2.8)	6	1.2	0.5–2.9	0.6 (0.0–106)
Non-current smokers without PHD (reference)	115	1.0			59	1.0		
Current smokers without PHD	27	0.9	0.6–1.3		25	1.6	1.0–2.5	
Non-current smokers with PHD	41	2.5	1.7–3.8		17	1.5	0.9–2.7	
Current smokers with PHD	5	1.7	0.6–4.4	0.5 (0.0–5.6)	4	1.2	0.4–3.5	0.2 (0.0–44)

OR, prevalence odds ratio; S, synergy index; CI, confidence interval; PHD, parental history of diabetes; BMI, body mass index.

No apparent interactions for a parental history of diabetes and smoking were observed either in men or in women (table 3). In analyses that excluded ex-smokers similar results were obtained (data not shown). No interactions between leisure time physical inactivity or physical inactivity at work and a parental history of diabetes were observed. Analyses that were based on combined information about leisure time physical inactivity and physical inactivity at work gave similar findings (data not shown). Among both men and women with a parental history of diabetes, there were very few smokers and very few individuals who reported a low level of physical activity. This explains the wide confidence intervals around the S scores.

## Discussion

In the present study a parental history of diabetes associated significantly with the presence of diabetes in men, whereas in women this association was less pronounced. Furthermore, in men but not in women a synergistic interaction between obesity and presence of a parental history of diabetes was observed.

The magnitude of the association between a parental history of diabetes and presence of diabetes in male offspring observed in our study agrees with findings from a large Swedish prospective registry-based study reporting a hazard ratio (HR) in men of 1.99 (95% CI 1.93–2.05) adjusted for age and socioeconomic status [24]. Our results in men also agree with those reported from a population-based cohort study performed in southern Germany reporting an age- and BMI-adjusted HR in men of 2.54 (95% CI 1.57–4.11). The weak association in women between a parental history of diabetes and presence of diabetes observed in our study does however not agree with findings in the two studies discussed above; both reported HR point estimates in women above 2.0 (after corresponding adjustments [24], [25]. Our result in women also deviates from findings in the Nurses’ Health Study cohort (NHS) including 73,227 American women. In the NHS, both a maternal- and a paternal history of diabetes were observed to increase the risk of developing type 2 diabetes; HR 1.85 (95% CI 1.70–2.10) and 1.78 (95% CI 1.61–1.95), respectively. The results were adjusted for age, smoking, physical activity and BMI among other factors. Although there is a major difference in magnitude of associations between our results in women and those reported from the NHS, we found a similar attenuation of the crude association when adjusting for smoking, BMI and physical activity. In the NHS the crude point estimates associated with both a maternal- and a paternal history of diabetes were above 2.0. Our data indicate that in women, more than in men, a parental history of diabetes may be explained by shared habits between parents and offspring.

Earlier studies analyzing family history of diabetes in relation to risk of type 2 diabetes in offspring have usually defined family history as presence of diabetes in either a parent or a sibling. Applying this wider definition in our study, the OR in women better agrees with earlier findings, e.g. those reported by Hilding et al. from a cross-sectional study based on data from a selection of participants included in the Stockholm Diabetes Prevention Program [12] where the OR after adjustment for age, smoking and level of physical activity was 1.7 (95% CI 1.0–3.0). A prospective study performed in Tromsø, Norway, however reported a HR in women of 2.44 (95% CI 1.83–3.26) [13] after multiple adjustments including BMI, leisure time physical inactivity and smoking. Our result in men when applying the wider definition of a family history of diabetes is somewhat less pronounced as compared to results earlier reported [13]. The InterAct study, a cohort study of genetic- and lifestyle factors influencing risk of type 2 diabetes including 27,779 men and women from eight European countries [11], [12] recently reported a crude HR of 2.64 (95% CI 2.22–3.14) in men and 2.77 (95% CI 2.49–3.07) in women associated with a family history of diabetes.

Our findings of an association between a family history of diabetes and presence of prediabetes in men are consistent with those of Hilding et al. reporting an OR adjusted for age, BMI and physical activity of 1.6 (95% CI 1.2–2.1) [12]. In women we noted no connection between a family history of diabetes and prediabetes, as opposed to Hilding et al. who associated a family history of diabetes with prediabetes in women, OR 1.5 (95% CI 1.1–2.1) [12]. One explanation for the discrepancy between study findings could relate to differences in definitions of prediabetes between the studies; in our study prediabetes was defined as IFG, whereas in the compared study prediabetes was defined as IFG or IGT (or a combination of these two). To the best of our knowledge, no other studies have investigated sex specific associations between a family history of diabetes in relation to risk of prediabetes. A study based on the Framingham cohort however reported an increased risk, in men and women combined, of presenting with a fasting plasma glucose level ≥6.1 mmol/l connected to a parental history of diabetes [14]. The effect observed was more pronounced for a maternal history of diabetes as compared to a paternal history (OR 3.0; 95% CI 2.2–4.2 vs. OR 1.8; 95% CI 1.3–2.7) – results that do not agree with our observations of similar results for maternal and paternal history of diabetes regarding association with prediabetes.

Our finding of a synergistic interaction in men for the combined exposure to parental history of diabetes and obesity regarding the development of diabetes is intriguing. If this finding reflects a true causal association, it would indicate that men who have a parental history of diabetes are more susceptible to the negative influence from obesity regarding the risk to develop diabetes. The interaction observed could be interpreted to reflect a gene-environmental interaction, assuming that BMI is mainly influenced by environmental factors such as dietary habits. However, BMI may also be influenced by genes indicating the possibility that also gene-gene interactions may contribute to our findings. The observation of an interaction between a parental history of diabetes and obesity concurs with results from a Canadian study, also cross-sectional in its design, suggesting that the combination of high BMI and a family history of diabetes increases the risk to develop diabetes [17]. However, the analyses were performed on men and women combined. Results from the InterAct study also indicate an interaction between obesity and family history of diabetes in men and women combined, with a more than 20-fold higher risk of type 2 diabetes in the double-exposed group as compared to the group of lean individuals without a family history [11]. Further support to our findings is provided from a study showing that BMI modifies the association between specific genetic variants, e.g. in the *PPARGC1A* gene, and glucose levels [26].

The use of SAD values in the analyses of interaction in men produced results that were more pronounced as compared to using BMI values, indicating that interaction effects may not be dependent on the distribution of body fat. The use of WHR values however produced weaker results as compared to both the use of SAD, BMI and waist circumference. One explanation for this observation may relate to that the WHR does not reflect abdominal adiposity to the same extent as do the other measures. Men with abdominal adiposity may be particularly susceptible to the influence from a parental history of diabetes. Interestingly, the genetic regulation of WHR has been suggested to differ from that of BMI [27] and waist circumference [28]. We speculate that the presence of potential gene-gene interactions may differ depending on body fat distribution.

The results from our analyses of interaction in women for the combination of a parental history of diabetes and high BMI are strikingly different as compared to those in men. Considering our finding suggesting a more pronounced effect from parental history of diabetes in men as compared to women regarding risk of diabetes, we speculate that genetic susceptibility to diabetes may be different in women as compared to in men. In support of this, a recently published meta-analysis reports identification of two loci showing sex differentiated association with diabetes [9]. We noted that a high BMI, in the absence of a parental history of diabetes, was more strongly associated with risk of diabetes in women than in men (table 3), perhaps indicating a different role of BMI in the etiology of diabetes in women as compared to men. In support of our speculations about sex specific etiology of diabetes, a study of 1221 European men and women showed that male offspring of type 2 diabetes patients more often than female offspring had impaired insulin sensitivity and beta cell function [29]. Further, it has been suggested that endogenous sex hormones may influence risk of type 2 diabetes differently in men and women [30]. Interestingly, exogenous sex hormones in postmenopausal women with coronary heart disease have been shown to reduce diabetes incidence with as much as 35% [31].

In neither men nor women interactions between a parental history of diabetes and physical inactivity or smoking respectively, were observed. The reason for these negative findings may be that there are no true effects, but our results must be interpreted with caution because the confidence intervals are very wide. Noteworthy, the number of individuals with combined exposure to a parental history of diabetes and smoking and physical inactivity, respectively, is low; perhaps a parental history of diabetes may bring about a healthier choice of life style.

Our finding that a maternal history of diabetes, as opposed to a paternal history, was more strongly associated with risk of diabetes in men is in agreement with earlier observations (in men and women combined) [11], [32]. Biologically, a stronger influence from maternal history may for example relate to changes in hormonal levels in the mother, due to stress and diet, affecting the fetus via epigenetic programming [33].

Our choice to use a parental history of diabetes as our main exposure variable, as opposed to a family history of diabetes was in part based upon earlier results suggesting that a parental history may better mirror genetic inheritance of diabetes phenotype than do family history. We also considered the methodological advantage of not including information on siblings related to the fact that differences in family size and age of siblings may bias results [34].

### Strengths and Limitations

Considering the high response rate in this study, the cohort participants should represent a cross section of the population of 60-year-olds in Stockholm.

In studies with a cross-sectional design a common limitation is an uncertainty whether exposure preceded the outcome or not. However, when studying genetic effects, or proxies for genetic effects, this is not an important limitation because the “exposure” is present from birth. However, the presence of overweight, physical inactivity and smoking may have varied over the years and it is possible that the measurements of these factors do not reflect the exposure as it was before the development of diabetes in study participants. Because a diagnosis with diabetes would in general be accompanied by strict advice about dietary habits (potentially influencing BMI), physical activity and smoking, all these exposures may have been underestimated in the group with diabetes, a situation that would involve a reduced efficiency of the confounding control and an underestimation of interaction effects.

A limitation with our diabetes classification is the non-separation of individuals with type 1 and type 2 diabetes. However, type 2 diabetes should account for a vast majority of the individuals identified with diabetes in our material, considering that type 2 diabetes is more common among the old. Data on age at diabetes onset in our material revealed that a disease onset earlier than 30 years of age was present only in 10 of the participants with diabetes. It should be noted however that 141 participants with diabetes out of 318 did not state their ageh at diabetes onset. Concerning parental history of diabetes, 8 mothers and 7 fathers were stated to have onset of diabetes before the age of 30. Out of 613 participants answering the question about diabetes in their parents, 417 did not state the year of diabetes onset for their father and 278 did not state what year their mother became affected. It would have been desirable in our study to distinguish between type 1 and type 2 diabetes because the disease processes differ. However, due to the large amount of missing data on age at diabetes onset we could not.

Parental history was measured using self-reported data. The influence of recall bias must therefore be considered. One possibility might be that individuals with diabetes better recall a positive parental history as compared to individuals without diabetes. Such a situation would give an overestimation of the results. Regarding prediabetes in our study, such recall bias is less likely assuming that the individuals with prediabetes were unaware of their elevated glucose levels. The accuracy of offspring’s reports of a parental history of cardiovascular disease and some of its risk factors have been examined [35] showing a high level of accuracy for diabetes with a negative predictive value (defined as the probability that the parent lack a parental history given a negative offspring report) greater than 90% in both men and women and a positive predictive value of 76% (95% CI 70%–82%) for men and 79% (95% CI 73%–85%) for women.

In conclusion, in men but not in women a parental history of diabetes was connected to diabetes in offspring. Moreover, in men a clear interaction between obesity and a parental history of diabetes was found. Provided that these results can be repeated and confirmed, it may be useful to target type 2 diabetes preventive measures specifically to obese men with a parental history of diabetes.
